# Dynamic Neuromagnetic Network Changes of Seizure Termination in Absence Epilepsy: A Magnetoencephalography Study

**DOI:** 10.3389/fneur.2019.00703

**Published:** 2019-07-02

**Authors:** Wenwen Jiang, Caiyun Wu, Jing Xiang, Ailiang Miao, Wenchao Qiu, Lu Tang, Shuyang Huang, Qiqi Chen, Zheng Hu, Xiaoshan Wang

**Affiliations:** ^1^Department of Neurology, The Affiliated Brain Hospital of Nanjing Medical University, Nanjing Medical University, Nanjing, China; ^2^Division of Neurology, MEG Center, Cincinnati Children's Hospital Medical Center, Cincinnati, OH, United States; ^3^Department of Neurology, The Affiliated Huai'an Hospital of Xuzhou Medical University, Huai'an, China; ^4^MEG Center, The Affiliated Brain Hospital of Nanjing Medical University, Nanjing, China; ^5^Department of Neurology, Nanjing Children's Hospital, Nanjing, China

**Keywords:** childhood absence epilepsy, magnetoencephalography, source localization, effective connectivity, cortico–thalamic network, seizure termination

## Abstract

**Objective:** With increasing efforts devoted to investigating the generation and propagation mechanisms of spontaneous spike and wave discharges (SWDs), little attention has been paid to network mechanisms associated with termination patterns of SWDs to date. In the current study, we aimed to identify the frequency-dependent neural network dynamics during the offset of absence seizures.

**Methods:** Fifteen drug-naïve patients with childhood absence epilepsy (CAE) were assessed with a 275-Channel Magnetoencephalography (MEG) system. MEG data were recorded during and between seizures at a sampling rate of 6,000 Hz and analyzed in seven frequency bands. Source localization was performed with accumulated source imaging. Granger causality analysis was used to evaluate effective connectivity networks of the entire brain at the source level.

**Results:** At the low-frequency (1–80 Hz) bands, activities were predominantly distributed in the frontal cortical and parieto–occipito–temporal junction at the offset transition periods. The high-frequency oscillations (HFOs, 80–500 Hz) analysis indicated significant source localization in the medial frontal cortex and deep brain areas (mainly thalamus) during both the termination transition and interictal periods. Furthermore, an enhanced positive cortico–thalamic effective connectivity was observed around the discharge offset at all of the seven analyzed bands, the direction of which was primarily from various cortical regions to the thalamus.

**Conclusions:** Seizure termination is a gradual process that involves both the cortices and the thalamus in CAE. Cortico–thalamic coupling is observed at the termination transition periods, and the cerebral cortex acts as the driving force.

## Introduction

Epilepsy is conventionally considered a functional brain disorder generated by excessive synchronization of large neuronal populations leading to a hypersynchronous state. Nevertheless, recent researches have demonstrated that synchronization increases at seizure onset and seizure termination, whereas it transiently decreases in between, which suggested that seizures are not so much a manifestation of hypersynchrony but instead courses of network reorganization (1–4). Furthermore, epilepsy is consequently formulated as a network disorder that constitutes aberrant circuits between the thalamic input and the cortex that are facilitating generalized spike-and-waves (SWDs) according to the most recent definition of the International League Against Epilepsy (ILAE).

Childhood absence epilepsy (CAE) is the most common pediatric epilepsy syndrome, occurring in 10–17% of all childhood onset epilepsy with a female preponderance (5). Typical absence seizures appear as brief disruptions of responsiveness without warning, interruptions of ongoing behavior that are difficult to notice. Electroencephalography (EEG) reveals characteristic bilateral, symmetrical, and synchronous discharges of 3 Hz generalized SWDs on a normal background activity (5, 6).

The SWDs pattern emerges suddenly and does not seem to be anticipated by specific EEG changes; thus, the common notion regarding CAE is that it is widespread and among generalized epilepsy. However, recent evidence has implied that it has focal origins, including cortical and subcortical sites (7–11). Brain functional connectivity studies have also indicated altered functional connectivity within the default mode network (DMN) and attentional networks in patients with absence seizures (8, 12, 13). Furthermore, the cortico–thalamo–cortical system has been observed to be responsible for the genesis of absence seizures according to animal models and human studies (14–17).

With decades of studies exploring SWDs formation, the underlying mechanisms for the spontaneous termination of absence seizures remains poorly understood, although knowledge of these intrinsic stopping mechanisms could potentially result in the development of novel therapeutic strategies.

The objective of the present study was to characterize the dynamic neuronal network at transitions from SWDs to post-SWDs in CAE. Magnetoencephalography (MEG), with a higher spatial resolution than EEG, enables the non-invasive assessment of neural activity measured on a submillisecond time scale (7, 11, 17–21), which makes it an ideal tool for investigating the network dynamics in CAE patients. In this work, the significant source localization and predominant effective connectivity (EC) network were investigated from 15 CAE patients at the source level in multi-frequency ranges with MEG.

## Materials and Methods

### Participants

Fifteen children (5–11 years old) with CAE were recruited from the Affiliated Brain Hospital of Nanjing Medical University and the Neurology Department of the Nanjing Children's Hospital from March 2015 to November 2017 (Table 1). Part of the patients in this article were overlapped with our previous study (17).

**Table 1:** Clinical characteristics of CAE patients.

**Patients**	**Duration of epilepsy (months)**	**Seizure frequency (times/day)**	**Seizure analyzed (times)**	**Ictal duration (s)**
1	48	8	2	20.1
2	2	8	1	14.4
3	3	20	4	8.7
4	8	15	2	10
5	10	4	1	5.5
6	5	2	2	24
7	11	4	2	14.2
8	12	6	3	14.1
9	22	6	1	10.6
10	72	12	1	17.1
11	4	5	1	25.5
12	63	3	2	7.05
13	1	10	4	12.5
14	4	10	3	14.5
15	6	5	4	8.4
Total (Mean)	18.1	8	33	13.8

Similar to our prior studies (17, 21, 22), the inclusion criteria were as follows: clinically diagnosed CAE consistent with the ILAE Proposal for Revised Classification of Epilepsies and Epileptic Syndromes; clinical EEG recordings with bilateral synchronous symmetrical 3 Hz SWDs on a normal background with at least one burst that lasted ≥4 s; normal development, normal neurological examination and normal brain magnetic resonance imaging (MRI); and <5 mm head movement during MEG recordings. The exclusion criteria were as follows: the presence of metal implants, such as cochlear devices and pacemakers, which will significantly interfere with MEG data; a history of seizures other than absence seizures or other clinically significant diseases; or unable to keep head still during MRI scans and/or MEG recordings.

This research was carried out in accordance with the recommendations of the ethical boards of the Affiliated Brain Hospital of Nanjing Medical University, Nanjing Children's Hospital and Nanjing Medical University. The protocol was approved by the ethical boards of the Affiliated Brain Hospital of Nanjing Medical University, Nanjing Children's Hospital and Nanjing Medical University. Informed consent was obtained from all children and their parents (guardians). All participants (guardians) gave written informed consent according to the Declaration of Helsinki.

### MEG Recording

The MEG data were recorded in a magnetically shielded room using a whole-head CTF MEG system with 275 channels (VSM Medical Technology Company, Canada) at the MEG Center of the Affiliated Brain Hospital of Nanjing Medical University.

Prior to data acquisition, three small coils were attached to the left and right pre- auricular points and nasion of each subject to measure head positions relative to the MEG sensors. The system allowed for head localization at an accuracy of 1 mm, and the head movement was limited to 5 mm during each recording. If head movement during a scan was >5 mm, the dataset was disregarded, and a new scan was recorded. All MEG data were acquired with noise cancellation of third-order gradients. The sampling rate was 6,000 Hz. During the MEG recordings, the participants were placed in a supine position, with their eyes slightly closed and remaining still (avoid swallowing or teeth clenching). An audio-visual system was utilized to monitor the subjects during the recording. For each patient, at least five epochs with a duration of 2 min were collected. If no SWDs were observed in the MEG signals of the first three recorded files, the participants were instructed to hyperventilate to provoke seizures. Furthermore, we routinely recorded one MEG dataset without a patient immediately before the experiment to identify system and environmental noise.

### MRI Scan

MRI scanning was performed on nine participants with a 3.0 T scanner (Siemens, Germany), whereas a 1.5 T scanner (Sigma, GE, USA) was used in six subjects. To facilitate the co-registration of MEG and MRI data, three fiduciary marks were placed in identical locations to the positions of the three coils used during the MEG recordings. All anatomical landmarks digitized in the MEG study were rendered identifiable in the MR images.

### MEG Data Analysis

#### Data Preprocessing

A bandpass filter of 1–4 Hz was applied for all MEG data to distinguish ictal segments from interictal segments without noise or artifacts because the ictal segments recorded during absence seizures are characterized by 3 Hz SWDs in MEG waveforms.

**Figure 1:**
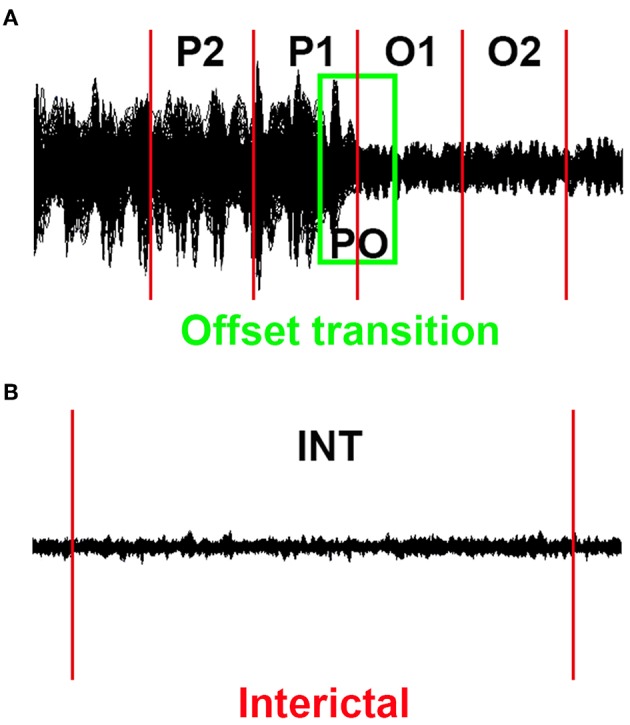
Schematic of MEG waveforms. Five 1 s offset transition epochs (P2, P1, PO, O1, and O2) for each epileptic activity **(A)** and the corresponding 100 s interictal epochs **(B)** were selected.

Given that epileptic events may be dynamic, we conducted the source localization and dynamic EC networks for four successive short time epochs. The end of the final slow-wave of the discharge was defined as the termination point (23, 24). Similar to our previously published research (17), successive epochs of 1 s were selected, with the total time window ranging from the period of 2 s prior to the discharge offset to 2 s following the offset (−2 s to +2 s, referred to as the transition period), and these successive epochs were referred to as P2, P1, O1, and O2 (P is short for the period prior to the SWD offset, O is short for the SWD offset). Similarly, an overlapped epoch of a 1 s time window around the offset point (from −500 ms to +500 ms, named as PO) was analyzed (Figure 1A). For contrast, interictal segments (as long as 100 s) that did not contain visual rhythmic SWDs were regarded as the control, the distances between which to the ictal period were at least 60 s (Figure 1B).

All data were filtered with bandpass filters at predefined frequency bands of 1–4 Hz (delta), 4–8 Hz (theta), 8–12 Hz (alpha), 12–30 Hz (beta), 30–80 Hz (gamma), 80–250 Hz (ripple), and 250–500 Hz (fast ripple). An example of HFO in both raw and filtered data can be seen in Supplementary Figure S1. Notch filters for 50 Hz, as well as the harmonics, were used to eliminate power-line noise.

#### Source Localization

Accumulated source imaging (ASI) was used to localize the significant neuromagnetic activity, which was defined as the volumetric summation of the source activity over a period of time and was specifically developed and optimized to analyze activities in CAE patients (17, 20, 25, 26). It can be described using the following equation:

(1) $$\begin{array}{r} Asi\left(r,s\right)=\underset{t=1}{\overset{t=n}{\sum }}Q\left(r,t\right) \end{array}$$

In Equation (1), *Asi* represents the accumulated source strength at location *r* and is computed at the specific frequency band *f*; *s* indicates the time slice; *t* indicates the time point of the MEG data within a selected slice *s*; *n* indicates the total time points of the MEG data; and *Q* indicates the source activity at source *r* and at time point *t*. We defined that *s* ≥ 1 and *s* ≤ *n*/2. The whole brain was scanned at 6 mm resolution (~17,160 voxels/sources).

The source activity was calculated using two-step beamforming (26). Specifically, the lead fields were computed for each source (or voxel position) to generate matrices with MEG data. To minimize the effect of coherent sources in the source localization, the sensors for partial sensor coverage for each voxel with lead field, referred to as voxel-based partial sensors, were selected (27). The covariance for the voxel-based partial sensors and two sets of magnetic source images using a vector beamformer were subsequently computed (27). The following step was to estimate the coherent source and the source orientation via the covariance matrix vector beamformer. Once the source orientation was determined, the source activity (or virtual sensor waveform) with a scalar beamformer was generated (27). Detailed mathematical algorithms and validations have been described in recently published papers (20, 26).

#### Estimating EC Networks

Based on previous studies (20, 27), virtual sensor waveforms for each source were calculated to analyze EC networks using correlation analysis and Granger causality (GC) at the source level. The source neural networks were estimated by analyzing the signal correlation of every pair of virtual sensors in selected time-windows of interictal, pre-offset, and offset periods (27, 28). To avoid focal connection caused by local volume conduction effects or the diffusiveness of sources, sources closer than 10 mm were considered one source. Specifically, we analyzed the relationship of virtual sensor signals from the two-source pair statistically by computing a correlation factor (or correlation coefficient). The correlation factors can be described as the following formulas:

(2) $$\begin{array}{r} R\left(x_{a},x_{b}\right)=\frac{C\left(x_{a},x_{b}\right)}{Sx_{a}Sx_{b}} \end{array}$$

where *R(*x_*a*_, x_*b*_*)* indicates the correlation of a source pair in two locations (“*a*” and “*b*”). The x_*a*_ and x_*b*_ indicate signals in two sources, which were paired for computing connection. *C(x*_*a*_, *x*_*b*_*)* represents the mean of the signals in the two sources. *Sx*_*a*_ and *Sx*_*b*_ indicate the standard deviation of the signals from the two sources. To avoid a potential bias, all possible connections for every two-source pair were computed with source-level analyses. The distribution of the EC for each possible pair of all voxel-based virtual sensors was co-registered to individual participant MRIs (20, 27).

According to the core idea that the cause precedes its effect, multivariate Granger causality was used to identify the direction of the connection. Specifically, a variable *Yi* Granger causes another variable *Yj* of the same process if the knowledge of *Yi*'s past improves the forecast of *Yj* (29–31). Similar to previous report (17), possible delays of source activities in 1–20 ms (1, 5, 10, 15, 20 ms, respectively) for 9 datasets were analyzed. Our pilot data showed that the delay of 10 ms generated reproducible results for datasets that were obtained from a group of patients at two or more time points (repeat measurements). Reproducible results mean the similarity of the results of the datasets obtained at different time for a same group of subjects and for different subjects. Therefore, if one source activity could forecast another source activity by a 10 ms time delay, the two sources were considered to be linked as a network and the prior source drove (or led) the posterior source (**Figure 4**). Otherwise, the two sources were not linked.

In the present study, correlation analysis and Granger causality were both used. The correlation is a statistical association, which refers to the degree to which a pair of two sources are linearly related. Granger causality is a statistical concept of causality that is based on prediction. The combination of correlation and Granger causality is to shift the time of one of the two sources and then calculating the correlation. The strong ECs was determined according to the strength of correlation. The first strong EC was defined as the predominant EC, and the direction of it was considered to be the directionality of the connection. We defined a threshold as a checkpoint to ensure the quality of the data. To statistically determine ECs, *t* values were calculated for all source pairs.

(3) $$\begin{array}{r} Tp=R\sqrt{\frac{K-2}{1-R^{2}}} \end{array}$$

In Equation (3), *Tp* indicates the *t* value of a correlation; *R* indicates the correlation of a source pair; *K* represents the number of the data points within a time window for computing the connection. We used the *Tp* value that had a corresponding *p*-value <0.01 as the threshold for obtaining the EC network and by adjusting thresholds from large to small, the first strong EC was identified.

In this study, inhibitory connection indicated increase of activation of one node resulted in the decrease of another node. Excitatory connections indicate increase of activation of one node resulted in the increase of another node. Blue and red were used to represent inhibitory and excitatory connections, respectively (**Figure 4**). Notably, magnetic source imaging-based neural networks were visualized in axial, coronal, and sagittal views to analyze the source connections.

The same data analyses were also applied to the MEG data obtained from the 20 healthy subjects. Based on previous report (32), neuromagnetic signals evoked by electrical stimulation were applied in our previous MEG study (17). MEG Processor software (https://sites.google.com/site/braincloudx/) was used to complete the aforementioned algorithms.

### Statistical Analysis

Group analyses of accumulated neuromagnetic source location and predominant EC among the interictal, pre-offset, and offset periods were implemented in seven frequency bands. The regions which displayed strongest source locations and the predominant EC were used for the group analysis. Fisher's exact test (chi-squared test) was performed on comparisons between the interictal and transition periods of seizures. The directionality of the predominant EC around the seizure offset was also analyzed. The threshold of statistical significance for differences was set at *p* < 0.05 for each test. The differences were corrected for multiple comparisons using the false discovery rate (FDR) approach:

(4) $$\begin{array}{r} pFDR=p_{s}*i/N \end{array}$$

where *p*_*s*_ equals 0.05; *N* represents the number of tests; *i* refers to the ranked index of the computed *p-*values (*pi*) (*i* of the minimal *p*-value is 1; *i* of the maximal *p*-value is *N*; and if there are several identical *p*-values, the *i* is the average value of these ranked indexes). If *p*_*i*_ < *pFDR*, the p_*i*_ is statistically significant. All statistical analyses were performed with SPSS 24.0 for Windows (SPSS Inc., Chicago, IL, USA).

## Results

### Participants

The gender ratio of the patients in the current study was 4:11 (male: female). For all subjects, the onset of symptoms occurred prior to the age of 10 years old. Forty-three seizures were captured during MEG recording; however, only 33 seizures were eligible for the subsequent analysis (removing the shorter, incomplete and inappropriate seizures), with an average ictal duration of 13.8 s (range: 5.5–25.5 s). Fifteen clean interictal MEG segments from the patients were selected and analyzed as the control. The details of the clinical data are presented in Table 1.

### Source Localization

**Figure 2:**
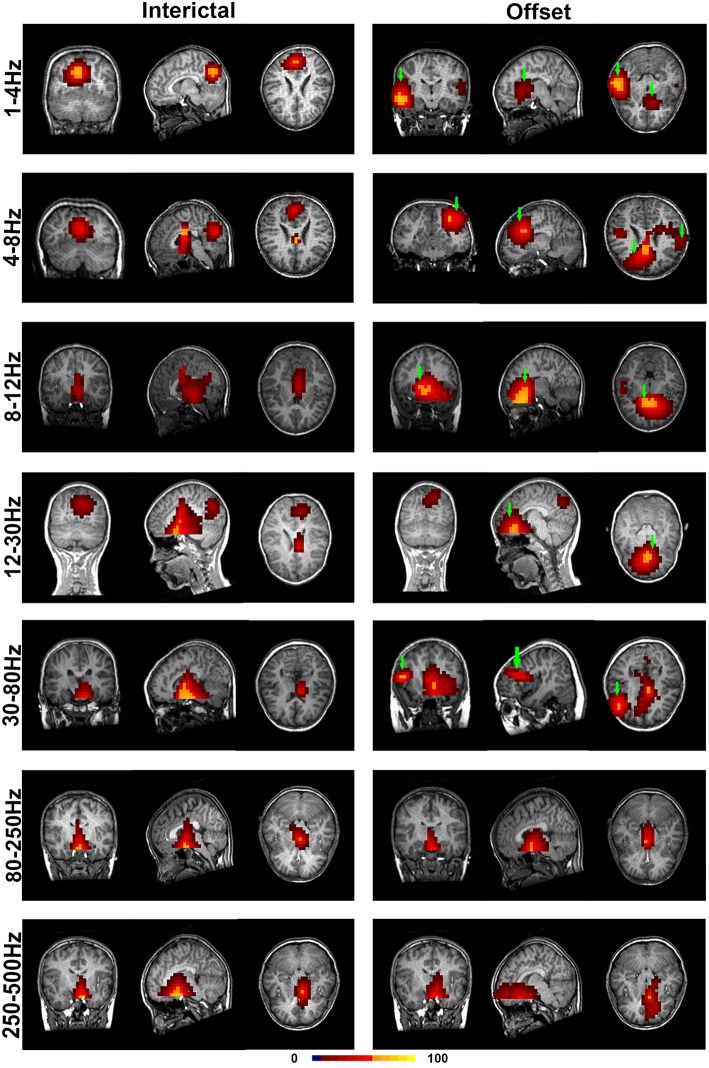
Accumulated source images of the interictal and termination transition periods showing spatial activities in seven pre-defined frequency bands recorded from CAE subjects. In comparison to the interictal periods, significantly altered patterns of source imaging were observed during termination transition periods in five lower frequency bands. Green arrows point toward the regions that show the activation only in the offset transition periods but not the interictal periods. The color bar indicates the color coding of the level of activation.

With co-registration to each subject's brain MRI for all selected MEG data, the regions of significant neuromagnetic activity in the interictal (INT) and termination transition periods of absence seizures (P2, P1, PO, O1, and O2) were obtained (Figure 2).

**Figure 3:**
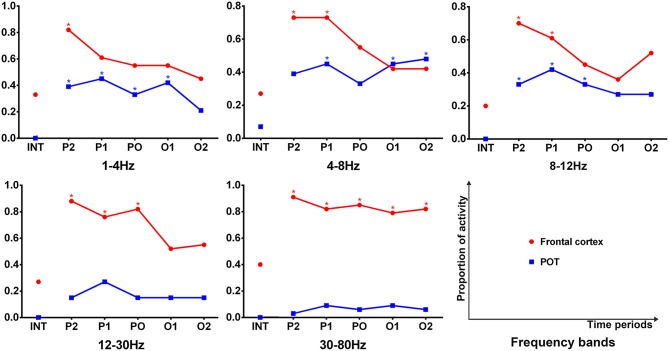
Comparison in sources of the frontal cortex and parieto–occipito–temporal junction (POT) between the interictal and offset transition periods for five lower frequency bandwidths. The y-axis indicates the proportion of data segments where the corresponding area was activated. Six time epochs are listed on the x-axis. Fisher's exact test was performed for comparisons between the termination transition (P2, P1, PO, O1, and O2) and interictal (INT) periods. Asterisks: *p* < 0.05 after FDR (corrected for 5 × 5 × 2 tests) with red for the frontal cortex and blue for the POT.

It was shown that each source image typically had 1–3 sources, which were very strong in comparison to the rest of the brain activity. It seemed that the areas estimated by high-frequency signals were much smaller when compared with the regions activated at the lower frequency bands. The analysis of the low-frequency bands indicated that epileptic activities had a tendency to localize in the frontal cortex (Supplementary Table S2) and parieto–occipito–temporal junction (POT) (Supplementary Table S3) during the termination transition periods as compared with the interictal periods (Figure 2). Both of the regions exhibited higher odds of activity during seizures at the delta (1–4 Hz), theta (4–8 Hz), and alpha (8–12 Hz) bands. Source localizations of aberrant activities at the beta (12–30 Hz) and gamma (30–80 Hz) bandwidths were more identified in the frontal cortex, particularly the medial frontal cortex. Detailed statistical results for these bands are shown in Figure 3. Nevertheless, for the high-frequency oscillations (HFOs, 80–500 Hz), sources predominantly located to the medial frontal cortex and deep brain areas (mainly the thalamus). No significant differences were identified in terms of the odds ratio of activity in the above brain areas among the six time periods.

These were the typical features of neuromagnetic source localization as described above. However, some sources at the seven predefined bandwidths were observed in the occipital cortex, temporal cortex, and parietal cortex in both the interictal and the termination transition periods (Supplementary Tables S4–S6). We did not find any significant differences between them. Even though source imaging may vary among seizures, it seemed that there is consistency among different seizures in each patient, which means that the inter-subject variation is more obvious than the intra-subject variation. It is possible that this variation may eventually explain genetic differences within this population or distinguish subgroups with specific co-morbidities in CAE.

### EC Networks

**Figure 4:**
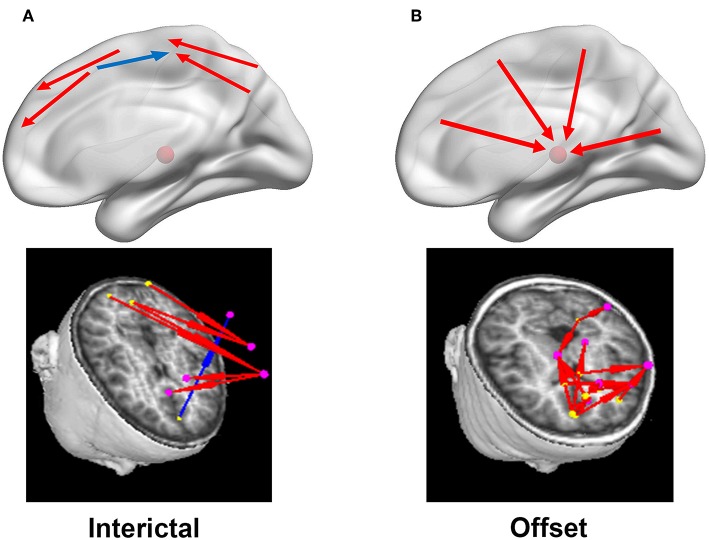
Diagrammatic drawings and examples of typical predominant EC networks in the interictal **(A)** and offset transition periods **(B)**. In the left column **(A)**, the connections were mainly identified between cortical sources in the interictal periods, with both positive (red arrows) and negative (blue arrows) connections. Nevertheless, strong positive cortico-thalamic connections were observed in the right column **(B)** for the termination periods, and the cortex primarily drove the thalamus (shown with the red schemaball above) during seizures. Yellow dot: the driving source. Pink dot: the source being driven.

**Figure 5:**
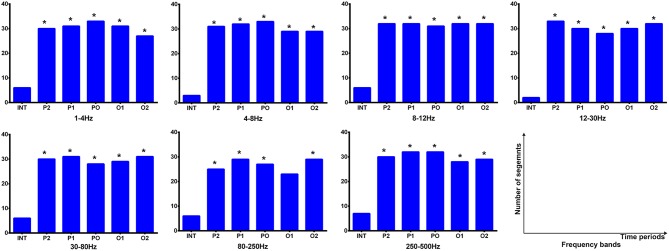
Cortico–thalamic EC during interictal and offset transition periods in seven frequency bands. The y-axis indicates the numbers of segments (total 33 segments for the offset transition periods, which represents 33 seizures analyzed from 15 CAE patients; total 15 segments for the interictal periods indicating 15 interictal MEG segments for control) where cortico–thalamic EC exists during corresponding periods. For all frequency bands, the transition (P2, P1, PO, O1, and O2) and interictal (INT) periods showed significant differences (marked with asterisks). *p < 0.05 after FDR (corrected for 7 × 5 tests).

During the interictal periods, EC networks were primarily observed between various cortical sites (e.g., the frontal and parietal cortices), whereas the deep brain areas were minimally involved. Both excitatory and inhibitory connection were detected. However, the cortices and deep brain areas (mainly the thalamus) showed a strong positive homogeneous EC in all analyzed frequency bands during the termination transition periods, especially P2, P1, and PO. We did not find any significant differences of EC networks in various frequency bandwidths. Schematic and representative examples of typical predominant EC networks in both interictal and offset transition periods from subjects with CAE are shown in Figure 4. As for the number segments of strong positive cortico–thalamic EC in the offset transition periods, it was larger than in the interictal periods (Figure 5, Supplementary Table S7).

**Figure 6:**
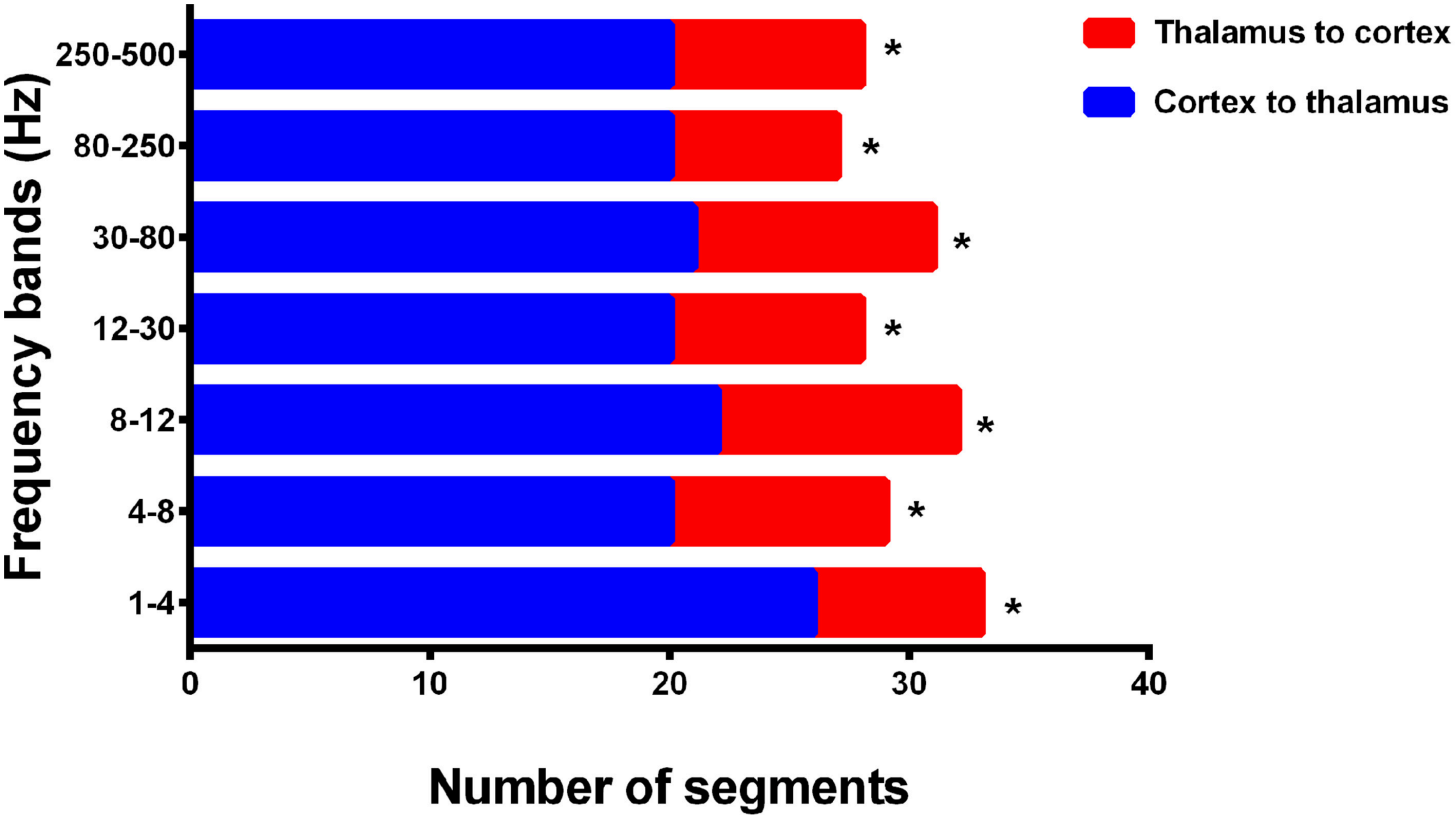
Directionality of cortico–thalamic connection in all seven frequency bands. The numbers of segments (total 33 segments representing 33 seizures analyzed from 15 CAE patients) where cortico–thalamic connection is observed during corresponding periods are displayed on the x-axis, whereas the y-axis indicates seven frequency bands. Blue rectangles: the direction of cortico–thalamic connection is from cortex to thalamus. Red rectangles: the direction of cortico–thalamic connection is from thalamus to cortex. *p < 0.05 after FDR (corrected for 7 tests).

In addition, we evaluated the directionality of the first strong positive cortico–thalamic EC in the termination transition periods and noted that the connections during SWDs in all seven analyzed frequency bands were mainly from cortical regions to the thalamus (*p* < 0.05) (Figure 6, Supplementary Table S8). The thalamus was always the ending point, whereas the locations of the cortices that drove connections varied among epileptic attacks, but sometimes were nearby between seizures in one participant.

## Discussion

There are a large body of articles on the mechanisms of initiation and generalization of discharges (8, 11, 19, 21, 33), with relatively few articles regarding the termination processes. To the best of our knowledge, this study is the first investigation to directly assess the source localization and predominant EC network of seizure termination using a multi-frequency analysis with MEG in CAE patients.

In our study, we detected evident frontal cortex source location during offset transition periods in five low-frequency bands (1–4, 4–8, 8–12, 12–30, and 30–80 Hz), while the activity of POT was observed at the delta (1–4 Hz), theta (4–8 Hz) and alpha (8–12 Hz) bands in these periods. These findings are in strong concordance with our recent researches (17, 22) and other relevant publications on CAE (9, 11, 19, 34). The frontal cortex has been described to play a crucial role in initiating and propagating absence seizures, including scalp EEG (35), EEG–fMRI (36, 37), and MEG (7, 11, 17–19, 21, 22, 34) studies, which demonstrates that absence seizures are not truly “generalized,” but might also have localized precursors. The significance of the lateral prefrontal cortex at SWDs termination of absence seizures was also underscored in a recent EEG-fMRI study (23). The distinct parietal regions were thought to represent part of the posterior DMN which are associated with states of awareness (34). Of note, the source localizations do not necessarily mean the abnormal brain tissues, but point to the areas engaged in a dynamic network of discharge termination. These activated brain areas may play a pivotal role in supporting the oscillation of the epileptogenic network (38). Consequently, we suppose the cortices, particularly the frontal cortex, are the critical structures relevant for the termination of absence seizures.

The dynamics of EC networks at the seizure termination transition periods in CAE were investigated using MEG data in the present study. The cortices and the thalamus showed significantly strong positive EC in all analyzed frequency bandwidths during the transition periods of termination, and the direction was from the cortices to the thalamus. These findings are in good agreement with the view of the involvement of the cortico–thalamic circuits in absence epilepsy during the pre-SWD to SWD and SWD to post-SWD transition periods from preceding rat models and human studies (16, 17, 21, 24, 25, 37, 39–41). Phenomena of the cortices regulating the thalamocortical circuitry have also been reported in studies on absence seizure (15, 17, 24, 37, 41–43). We also observed strong EC several seconds following the offset, which might manifest an SWD re-initiation attempt (40). Moreover, a study of genetic absence epileptic rats using invasive recording electrodes emphasized increased coupling between distinct thalamic nuclei at the seizure termination transition periods (40). Nevertheless, as a result of the relatively low spatial resolution of MEG, we could not distinguish between different thalamic nuclei and regarded the thalamus in its entirety similar to other MEG and fMRI studies (17, 18, 21, 25, 34, 36). In this study, we have eliminated local volume conduction effects by taking the sources within 10 mm as one source, which would not yield any connectivity. In addition, we quantitatively analyzed the connectivity with Granger causality analysis and statistically analyzed the differences between phases/stage of seizures. We consider that remaining effects and spurious connectivity would existing in all stages that would not affect our main findings, which are the dynamic changes of network during the stages of seizures. Specifically, the time window was adjustable in our connectivity analysis and the delay was set to 10 ms which generated reproducible results for the present study. However, the time may depend on distance, type and density of a connection. Further verification is needed in the future.

In this study, we identified concordant spatial localization of the medial frontal cortex and thalamus for high-frequency ranges (80–500 Hz) at the offset transition and interictal periods. The overlapped areas of neuromagnetic source location between the interictal and ictal periods in high-frequency bands were observed in several EEG and MEG reports on epilepsy (17, 22, 44, 45), which indicated that the high-frequency interictal and ictal activities might be generated by the same brain areas. It seemed that even without capturing seizures, it is feasible to reliably deduce findings from a brief MEG recording of HFOs to a definitive characterization of epilepsy (44). Furthermore, Tenney et al. implicated that the ictal neuromagnetic sources were localized primarily in the frontothalamic regions at 70–150 Hz in subjects with CAE (34), which was in line with our findings. The viewpoint that slow oscillators can involve many groups of neurons over large brain areas, while fast oscillators with short duration time windows are better suited to local neighboring interactions could also be an explanation for our results on source location in high-frequency bands (34, 46).

It is known that mechanisms of seizure termination range from the scale of neuronal membranes and synapses to an intermediate scale comprising local neuron and interneuron networks, as well as a larger scale characterized by long-range cortico-subcortical modulating networks (47). The increase of synchrony was observed immediately before seizure termination (24, 42, 48, 49). Strong activation of sodium and calcium inflow contributes to overwhelming potassium conductances that may silence neuronal firing (42). Although the exact relationship between the increase of synchronization and seizure termination remains to be clarified, the highly synchronous paroxysmal activity in large neuronal networks detected at the end of a seizure may promote seizure termination, or at least represent a marker of mechanisms that mediate seizure termination (42, 49). The directed connectivity findings of the seizure termination period in the current study are consistent with the previously described view. Moreover, it was shown that synchronization increases slightly around the onset of seizure, followed by a decrease at the seizure state, and then increases dramatically immediately before seizure termination in several previous reports (1–4). The authors speculated that by simultaneously driving extended neuronal networks into a refractory state, this increase of synchronization might be a mechanism for the termination of ictal activity (50). Although it has been postulated that electrical stimulation should induce desynchronization on the assumption that seizures are due to hypersynchronous activity (51), our findings among other results demonstrate that the opposite may be the case. That is, an increase of synchronization could facilitate seizure termination.

Nevertheless, seizure termination, as a highly complicated process that is influenced and dependent on various effects, is far from being completely understood. Our motivation to investigate this intricate mechanism is based on the hope that a deeper understanding of it may potentially result in complementary insights into the brain autoregulatory mechanisms, which will provide new avenues for advances in the diagnostics and treatment of epilepsy.

Several limitations of the present study should be considered. First, it remains enigmatic to localize deep brain sources using the current MEG field with a relatively low spatial resolution. The deep brain activities detected in the study might be artifacts and noise, which may be projected to the center of the head and thus appear as thalamic activity. Although previous reports (32, 52) have demonstrated the probability that MEG can detect deep brain activity and we have done some studies with somatosensory evoked magnetic fields (17), more convincing data are necessary to completely rule out that deep brain HFOs are not artifacts or noise. Second, the number of patients and ictal seizures analyzed was limited. We applied strict criteria and excluded other forms of idiopathic generalized epilepsy, which made the patients in this study constitute a strictly homogeneous group in terms of diagnosis, despite the small size. However, different modalities of the absence seizure termination may exist in this population and the inter-individual variation was found to be clearly greater than the intra-individual variation. Further studies are required based on larger cohort sizes. Third, we merely focused on the increased positive EC between the cortices and the thalamus at the SWD offset and not on the decreased or negative EC in this paper, which may be related to these dynamic periods. Further explorations on decreased or negative connections are required. Fourth, the time epochs of one second selected in this study were not short enough in terms of seizure dynamics. Further analysis with shorter time epochs is necessary in our future work. Finally, the results of the current study may include artifacts from electromyography, magnetocardiography, and other signals. Specifically, MEG signals were recorded under the same experimental conditions, and accumulated technology was used. Although we have exerted considerable efforts to minimize these artifacts, further studies are necessary to ascertain whether artifacts have been completely eliminated.

## Conclusion

In the present study, we investigated the dynamics of the neuromagnetic network from the SWDs to post-SWDs transition periods in CAE patients and observed distinct neuromagnetic signatures compared with the interictal periods in both low- and high-frequency ranges with MEG that have not typically been reported. The cortices, including the frontal cortex and the POT, might be responsible for the termination of absence seizures. The directed connectivity analysis demonstrated that the cortico-thalamo-cortical network participates in the termination transition periods of absence seizures, and the cortices may exert control on the activity of the thalamus that ultimately leads to seizure termination. The changes of the networks at SWDs offset in CAE might suggest there is a pathologically predisposed state toward seizure termination with early sources, which demonstrates that seizure offset is a gradual process that builds up in a dynamic network. However, the current study is a preliminary study, and it would be interesting to further investigate the SWD termination or the entire ictal event of absence seizure with a larger data set in the future.

## Ethics Statement

This research was carried out in accordance with the recommendations of the ethical boards of the Affiliated Brain Hospital of Nanjing Medical University, Nanjing Children's Hospital and Nanjing Medical University. The protocol was approved by the ethical boards of the Affiliated Brain Hospital of Nanjing Medical University, Nanjing Children's Hospital and Nanjing Medical University. Informed consent was obtained from all children and their parents (guardians). All participants (guardians) gave written informed consent according to the Declaration of Helsinki.

## Author Contributions

WJ and XW designed the study. SH, LT, AM, and ZH recruited the subjects. QC recorded MEG data. WJ and CW analyzed the data. WJ, CW, and WQ wrote the manuscript, while JX and XW revised it. All authors signed the final approval for publication.

## Conflict of Interest Statement

The authors declare that the research was conducted in the absence of any commercial or financial relationships that could be construed as a potential conflict of interest.
