# Supplementary material for: Dynamic Neuromagnetic Network Changes of Seizure Termination in Absence Epilepsy: A Magnetoencephalography Study
Source: Front Neurol. 2019 Jul 2;10:703. doi: 10.3389/fneur.2019.00703 (PMC6626921; doi:10.3389/fneur.2019.00703)
Supplement: Supplementary file 1 [file Table_1.DOCX]

***Supplementary Materials***

**SUPPLEMENTARY FIGURE**


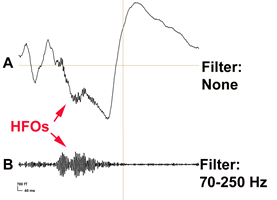


MEG HFOs are identifiable in raw data (“**A**”, no filtering). “False HFOs” resulted from filtering can be excluded (“**B**”).

**Legend S1:** An example of HFO in raw data (**A**) and filtered data (**B**). In the case of none filter used, both the true and false HFOs are identifiable in raw data (**A**). However, False HFOs resulted from filtering can be excluded, and the true HFOs are displayed clearly (**B**).

**SUPPLEMENTARY TABLES**

**Supplementary Table S2.** Source localization in the frontal cortex

|  | 1-4Hz | 4-8Hz | 8-12Hz | 12-30Hz | 30-80Hz | 80-250Hz | 250-500Hz |
| --- | --- | --- | --- | --- | --- | --- | --- |
| INT | 5 | 4 | 3 | 4 | 6 | 10 | 14 |
| P2 | 27 | 24 | 23 | 29 | 30 | 29 | 32 |
| P1 | 20 | 24 | 20 | 25 | 27 | 25 | 32 |
| PO | 18 | 18 | 15 | 27 | 28 | 26 | 30 |
| O1 | 18 | 14 | 12 | 17 | 26 | 24 | 28 |
| O2 | 15 | 14 | 17 | 18 | 27 | 25 | 32 |

**Legend S2:** Segments of **s**ource localization in the frontal cortex in interictal (INT) and offset transition periods (P2, P1, PO, O1, O2) (total 33 segments for the offset transition periods, representing 33 seizures analyzed from 15 CAE patients; total 15 segments for the interictal periods, which indicates 15 interictal MEG segments for control). At the low-frequency bands (1-80Hz), sources were localized in the frontal cortex during the termination transition periods as compared with the interictal periods (P<0.05). No significant differences were identified for HFO among the six time periods.

**Supplementary Table S3.** Source localization in the parieto–occipito–temporal junction (POT)

|  | 1-4Hz | 4-8Hz | 8-12Hz | 12-30Hz | 30-80Hz | 80-250Hz | 250-500Hz |
| --- | --- | --- | --- | --- | --- | --- | --- |
| INT | 0 | 1 | 0 | 0 | 0 | 0 | 0 |
| P2 | 13 | 13 | 11 | 5 | 1 | 0 | 1 |
| P1 | 15 | 15 | 14 | 9 | 3 | 1 | 0 |
| PO | 11 | 11 | 11 | 5 | 2 | 2 | 1 |
| O1 | 14 | 15 | 9 | 5 | 3 | 1 | 3 |
| O2 | 7 | 16 | 9 | 5 | 2 | 3 | 3 |

**Legend S3:** Segments of **s**ource localization in the parieto–occipito–temporal junction (POT) in interictal (INT) and offset transition periods (P2, P1, PO, O1, O2) (total 33 segments for the offset transition periods, representing 33 seizures analyzed from 15 CAE patients; total 15 segments for the interictal periods, which indicates 15 interictal MEG segments for control). It was shown that epileptic activities had a tendency to localize in the POT during the termination transition periods at the delta (1–4 Hz), theta (4–8 Hz) and alpha (8–12 Hz) as compared with the interictal periods (P<0.05). No significant differences were detected for HFO among the six time periods.

**Supplementary Table S4.** Source localization in the middle occipital cortex

|  | 1-4Hz | 4-8Hz | 8-12Hz | 12-30Hz | 30-80Hz | 80-250Hz | 250-500Hz |
| --- | --- | --- | --- | --- | --- | --- | --- |
| INT | 9 | 9 | 11 | 9 | 2 | 0 | 0 |
| P2 | 6 | 4 | 6 | 6 | 0 | 0 | 1 |
| P1 | 6 | 4 | 7 | 3 | 1 | 0 | 2 |
| PO | 5 | 4 | 3 | 4 | 1 | 0 | 0 |
| O1 | 4 | 5 | 10 | 9 | 2 | 1 | 0 |
| O2 | 7 | 10 | 12 | 8 | 3 | 0 | 1 |

**Legend S4:** Segments of **s**ource localization in the middle occipital cortex in interictal (INT) and offset transition periods (P2, P1, PO, O1, O2) (total 33 segments for the offset transition periods, representing 33 seizures analyzed from 15 CAE patients; total 15 segments for the interictal periods, which indicates 15 interictal MEG segments for control).

**Supplementary Table S5.** Source localization in the temporal cortex

|  | 1-4Hz | 4-8Hz | 8-12Hz | 12-30Hz | 30-80Hz | 80-250Hz | 250-500Hz |
| --- | --- | --- | --- | --- | --- | --- | --- |
| INT | 0 | 0 | 0 | 0 | 0 | 0 | 0 |
| P2 | 3 | 1 | 4 | 1 | 2 | 6 | 3 |
| P1 | 1 | 2 | 2 | 1 | 2 | 4 | 4 |
| PO | 4 | 5 | 3 | 3 | 2 | 8 | 1 |
| O1 | 4 | 4 | 3 | 2 | 0 | 5 | 4 |
| O2 | 5 | 3 | 4 | 1 | 2 | 4 | 3 |

**Legend S5:** Segments of **s**ource localization in the temporal cortex in interictal (INT) and offset transition periods (P2, P1, PO, O1, O2) (total 33 segments for the offset transition periods, representing 33 seizures analyzed from 15 CAE patients; total 15 segments for the interictal periods, which indicates 15 interictal MEG segments for control).

**Supplementary Table S6.** Source localization in the precuneus

|  | 1-4Hz | 4-8Hz | 8-12Hz | 12-30Hz | 30-80Hz | 80-250Hz | 250-500Hz |
| --- | --- | --- | --- | --- | --- | --- | --- |
| INT | 2 | 0 | 0 | 0 | 0 | 0 | 0 |
| P2 | 1 | 1 | 4 | 1 | 2 | 6 | 3 |
| P1 | 2 | 2 | 2 | 1 | 2 | 4 | 4 |
| PO | 1 | 5 | 3 | 3 | 2 | 8 | 1 |
| O1 | 0 | 4 | 3 | 2 | 0 | 5 | 4 |
| O2 | 5 | 3 | 4 | 1 | 2 | 4 | 3 |

**Legend S6:** Segments of **s**ource localization in the precuneus in interictal (INT) and offset transition periods (P2, P1, PO, O1, O2) (total 33 segments for the offset transition periods, representing 33 seizures analyzed from 15 CAE patients; total 15 segments for the interictal periods, which indicates 15 interictal MEG segments for control).

**Supplementary Table S7.** Cortico–thalamic effective connectivity (EC) during interictal, pre-offset and offset periods in seven frequency bands

|  | 1-4Hz | 4-8Hz | 8-12Hz | 12-30Hz | 30-80Hz | 80-250Hz | 250-500Hz |
| --- | --- | --- | --- | --- | --- | --- | --- |
| INT | 6 | 3 | 6 | 2 | 6 | 6 | 7 |
| P2 | 30 | 31 | 32 | 33 | 30 | 25 | 30 |
| P1 | 31 | 32 | 32 | 30 | 31 | 29 | 32 |
| PO | 33 | 33 | 31 | 28 | 28 | 27 | 32 |
| O1 | 31 | 29 | 32 | 30 | 29 | 23 | 28 |
| O2 | 27 | 29 | 32 | 32 | 31 | 29 | 29 |

**Legend S7:** Segments of cortico–thalamic effective connectivity (EC) during the interictal (INT) and termination transition periods (P2, P1, PO, O1, O2) (total 33 segments for the offset transition periods, representing 33 seizures analyzed from 15 CAE patients; total 15 segments for the interictal periods, which indicates 15 interictal MEG segments for control). The cortices and the thalamus showed strong positive homogeneous EC in all analyzed frequency bands during the offset transition periods, especially P2, P1 and PO, with the segments of strong positive cortico–thalamic EC in the offset transition periods larger than in the interictal periods. **p* < 0.05 after FDR (corrected for 7 × 5 tests).

**Supplementary Table S8.** Directionality of cortico–thalamic connection in all seven frequency bands

| Frequency band (Hz) | Cortex to thalamus | Thalamus to cortex | *P* value | FDR threshold |
| --- | --- | --- | --- | --- |
| 1-4Hz | 26 | 7 | 0.0000^*^ | 0.0003 |
| 4-8Hz | 20 | 9 | 0.0063^*^ | 0.0371 |
| 8-12Hz | 22 | 10 | 0.0031^*^ | 0.0218 |
| 12-30Hz | 20 | 8 | 0.0028^*^ | 0.0280 |
| 30-80Hz | 21 | 10 | 0.0066^*^ | 0.0334 |
| 80-250Hz | 20 | 7 | 0.0011^*^ | 0.0198 |
| 250-500Hz | 20 | 8 | 0.0028^*^ | 0.0280 |

**Legend S8:** Segments of directionality of cortico–thalamic EC network in all seven frequency bands during the offset transition periods (P2, P1, PO, O1, O2) (total 33 segments for the offset transition periods, representing 33 seizures analyzed from 15 CAE patients). The connections during SWDs in all seven analyzed frequency bands were mainly from cortical regions to the thalamus. ^*^*p* < 0.05 after FDR (corrected for 7 tests).
